# Whole genome sequencing-based analysis of genetic predisposition to adult glioblastoma

**DOI:** 10.1038/s41525-025-00526-z

**Published:** 2025-10-30

**Authors:** Mark P. van Opijnen, Devin R. van Valkengoed, Joep de Ligt, Filip Y. F. de Vos, Marike L. D. Broekman, Edwin Cuppen, Roelof Koster

**Affiliations:** 1 Department of Cell and Chemical Biology, Leiden University Medical Center, Leiden, the Netherlands; 2 Department of Neurosurgery, Leiden University Medical Center, Leiden, the Netherlands; 3 Hartwig Medical Foundation, Amsterdam, the Netherlands; 4 Department of Medical Oncology, Utrecht University Medical Center, Utrecht, the Netherlands; 5 Department of Neurosurgery, Haaglanden Medical Center, the Hague, the Netherlands; 6 Center for Molecular Medicine and Oncode Institute, University Medical Center Utrecht, Utrecht, the Netherlands; 7 The Netherlands Cancer Institute, Amsterdam, the Netherlands

**Keywords:** Cancer genetics, Cancer genomics, Cancer genetics, Cancer genomics

## Abstract

The germline genetic susceptibility to adult glioblastoma remains unclear. With the option of broad molecular testing, it is crucial that clinicians are aware of the a priori probability of finding germline predisposition in glioblastoma patients. Here, we studied the genetic predisposition to adult glioblastoma using paired tumor-normal WGS data in an unselected, average cohort of 92 glioma WHO grade 4 patients. In 10 patients (11%), 12 Pathogenic Germline Variants (PGVs) were found in genes strongly associated with familial glioblastoma (*MSH6* (3x), *PMS2* (5x), *MSH2*, *NF1*, *BRCA1*) or medulloblastoma (*SUFU*). In six of these patients (60%), causality was supported by a second (somatic) event and/or a matching genome-wide mutational signature. Thus, germline predisposition does play a role in the development of adult glioblastoma, with mismatch repair deficiency being the main mechanism. Our results also highlight the benefits of tumor-normal WGS for glioblastoma patients and their families, beyond identifying actionable mutations for therapy.

## Introduction

Glioblastoma, a primary brain tumor, is the most common and most aggressive malignant brain tumor in adults. Despite intensive treatment consisting of surgical resection followed by radiotherapy with concurrent and sequential chemotherapy, the prognosis remains poor with a median survival of 15 months^1,2^. One of the contributing factors challenging effective treatment strategies is the inter- and intratumoral heterogeneity of this devastating disease^3^. This also becomes apparent in the complexity revealed by genomics^4^ and single-cell RNA sequencing^5,6^. Nevertheless, genomic analysis of the tumor is considered a promising technological development that could enable personalized treatment strategies. The most comprehensive approach for genomic analysis is whole-genome sequencing (WGS), which has been clinically validated for diagnostic purposes^7,8^. WGS is not yet widely used in routine settings, especially for glioblastoma, mostly because of a lack of evidence of clinical utility, costs, or both. Therefore, we have initiated the GLOW trial, a clinical study to explore the potential added value of WGS for recurrent glioblastoma patients^9^. As WGS analyses typically include a control normal DNA sample (e.g., from blood) to distinguish somatic variants (acquired in the tumor cell) from germline variants (inherited), they may also reveal potential genetic predisposition to glioblastoma. This knowledge might be relevant to patients and their relatives, and the presence of familial predisposition is often an important question in the consulting room. Furthermore, variants in several predisposition genes are increasingly important for (immune- or targeted) therapy selection^10–16^.

In contrast to other (sub)types of cancer, for instance breast cancer and colon cancer, but also to pediatric gliomas, the prevalence of heredity in adult glioblastoma patients is still largely unexplored, mainly due to lower incidence and limited datasets that are available to investigate this topic^17–19^. In general, an estimate of approximately 5% of all glioma patients have a positive family history for glioma, with twofold to elevenfold increased incidence ratios in those families^20–22^. These cases show similarity to sporadic cases in terms of demographics (age, gender), morphology and tumor grade, and the penetrance of hereditary glioma is suggested to be low^23^. Hereditary glioblastoma, also called familial glioblastoma, caused by single-gene hereditary disorders, is very rare^24^ and often involves predisposition of a range of tumor types. Current knowledge is limited to a few syndromes including neurofibromatosis type 1 (*NF1* mutation, autosomal dominant), Li-Fraumeni syndrome (*TP53* mutation, autosomal dominant), Turcot syndrome type 1 (mismatch repair genes [*MLH1*, *MSH2*, *MSH6* and *PMS2*] mutations, autosomal dominant) and Lynch or constitutional mismatch repair deficiency (mismatch repair genes mutations, autosomal dominant [Lynch] or recessive [constitutional mismatch repair deficiency])^25–27^. Furthermore, in enriched cohorts (i.e., selected for personal and/or family history) pathogenic variants in *BRCA1* and *2*, *CHEK2*, *HERC2*, *MUTYH*, *NF1*, *POT1* and *TERF2* have been associated with glioblastoma^20,28–30^, although their contribution to glioblastoma development remains unclear, since second-hit somatic variants were not observed for many^29^. Apart from these syndromes, familial glioblastoma is thought to be multifactorial and autosomal recessive^31–33^. Genome-wide association studies have identified several risk loci for glioblastoma, but the causality of specific variants or genes in these regions remains unclear^24,34^.

Taken together, in sporadic and/or late-onset glioblastoma cases, the prevalence and contribution of pathogenic germline variants (PGVs) remain unclear. It is, therefore, of interest to systematically analyze the complete germline genome of unselected glioblastoma patients, including small and structural variants, to identify genes with PGVs as potential candidates for cancer predisposition. This study thus aimed to gain novel insight into the prevalence of genetic predisposition to glioblastoma by retrospectively analyzing germline data of an unselected, adult glioblastoma patient population.

## Results

### Patient characteristics

A total of 98 patients met the inclusion criterion of ‘adult glioma WHO grade 4’ and were included in this study for germline predisposition analysis. Of these, 70.4% (69/98) were male, and the median age for males and females was 61 years. Most of the patients had a primary, isocitrate dehydrogenase (*IDH*) wildtype glioblastoma (93.9%, 92/98), while 6.1% (6/98) of the tumors had a somatic *IDH* mutation (classifying them as astrocytoma WHO grade 4). These six patients with an *IDH1/2* mutation were excluded from further analysis. The family history, particularly regarding the occurrence of malignant neoplasms, was unidentified.

### Germline findings in an average glioblastoma population

After filtering for canonical coding effects, gnomAD population frequency and quality score, a total of 402 small variants and five structural variants (SVs) were detected in 107 of the 170 different genes. Filtering for variants that were annotated as ‘(likely) pathogenic’ or ‘unknown’ in ClinVar following manual curation resulted in a total of 29 (including three SVs) PGVs in 17 different genes in 24 unique patients (26% of all patients). Of these 29 PGVs, 11 were observed in genes with an explicitly recessive inheritance and 18 in genes having dominant inheritance. All 11 PGVs in recessive genes were monoallelic and, therefore, excluded from overall prevalence, because only biallelic or compound heterozygous germline variants in such genes are considered as having associated hereditary risks (Table 1).

**Table 1:** Findings after manual curation of possibly interesting variants

Patient	Gene	c.HGVS^1^	p.HGVS^2^	Classification	Inheritance	Mechanism	Tumor status	Category	TMB	MSI	Somatic drivers	PreTreatment
HMF003436A	*MSH6*	c.467 C > G	p.Ser156*	P	dominant	LOF	MSI & second hit(s) c.1444 C > T, p.Arg482* c.3254delC, p.Phe1088fs	1	**37.9**	**7.8**	*ATM, CDKN2A*, *MSH6*, *TP53*, chr10 loss	No
HMF002509A	*PMS2*	c.1882C>T	p.Arg628*	P	dominant	LOF	MSI & second hit(s) c.943 C > T, p.Arg315* c.1732 C > T, p.Arg578Cy	1	**339**	**39**	PTEN, PMS2, POLE	Yes
HMF003795A	*BRCA1* *SDHA*	c.2210delC c.91 C > T	p.Thr737fs p.Arg31*	P P	Dominant dominant	LOF LOF	LOH, HRD signature no second hit	1 3	8.6	0.2	TERT, TP53, PTEN, chr7 gain, chr10 LOH	Yes
HMF001910A	*PMS2*	c.137 G > T c.736_741delinsTGTGTGTGAAG	p.Ser46Ile p.Pro246fs	LP P	dominant	LOF	MSI	1	**11.7**	**6.4**	TP53, VHL, CDKN2A del	No
HMF000729A	*PMS2*	c.325dupG c.825 A > G	p.Glu109fs p.Gly275= (splice)^5^	P P^5^	dominant	LOF	MSI	1	**279**	**48.2**	ATRX, PTEN, TP53	n/a
HMF007273A	*MSH6*	c.742delC	p.Arg248fs	P	dominant	LOF	MSI, LOH	1	**89**	**2.6**	ATRX, PTEN, RB1, TERT, TP53, chr10 LOH, chr7 gain	Yes
HMF002821A	*SUFU*	c.436 C > T	p.Arg146*	P	dominant	LOF	LOH	2	2.1	0.1	ATRX, TP53, 10q loss/LOH	No
HMF006763A	*NF1*	complex event^&^ (see below)	unbalanced	P	dominant	LOF	no second hit^#^	2	3.5	0.1	TERT, EGFR gain, chr7 gain, chr10 LOH	Yes
HMF005706A	*MSH6*	c.3514dupA	p.Arg1172fs	P	dominant	LOF	MSS, no second hit	2	3.2	0.1	TERT, CDKN2A, EGFR, PTEN, chr10 loss/LOH, chr7 gain	No
HMF000788A	*MSH2*	c.942+2delT	p.? (splice?)^6^	LP^6^	dominant	LOF	MSS, no second hit variant lost in tumor	2	3	0.1	NF1, TERT, CDKN2A, MET, chr7 gain, chr10 loss/LOH	Yes
HMF000649A	*BUB1B*	c.2210 T > G	p.Leu737*	P	recessive	LOF	second hit, large deletion including *BUB1B*	3	3.7	0.1	CDKN2A, EGFR, chr7 gain, chr10loss/LOH	No
HMF000655A	*ATR*	Inversion (see below)	Inversion exon 42-47 + XRN1	P	dominant	LOF	no second hit	3	4.4	0.2	EGFR, TERT, CDKN2A, chr7 gain, chr10 loss/LOH	Yes
HMF000925A	*CHEK2*	c.1229delC	p.Thr410fs	P	dominant^±^	LOF	no second hit	3	3.5	0.1	PTEN, TERT, CDKN2A, EGFR, chr10 loss/LOH	Yes
HMF000842A	*CHEK2*	c.1229delC	p.Thr410fs	P	dominant^±^	LOF	no second hit	3	2.7	0.1	EGFR, TERT, CDKN2A, chr7 gain, chr10 loss/LOH	No
HMF007010A	*CHEK2*	c.1229delC	p.Thr410fs	P	dominant^±^	LOF	no second hit	3	4	0.1	PTEN, TERT, TP53, RB1, chr7/10 loss/LOH	Yes
HMF001235A	*ERCC3*	c.760 C > T	p.Gln254*	P	recessive	LOF	no second hit	3	2.5	0.1	TERT, CDK4, PTEN, TERT, chr7 gain, chr10 loss/LOH	No
HMF001434A	*BLM*	Deletion (see below)	deletion exon 2-22	P	recessive	LOF	no second hit	3	3	0.1	EGFR, TERT, CDKN2A, chr7 gain, chr10 loss/LOH	No
HMF003406A	*BLM*	c.3558+1 G > T	p.? (splice)	P	recessive	LOF	no second hit	3	2.9	0.1	NF1, PTEN, TERT, CDKN2A, EGFR, chr7 gain, chr10 loss/LOH	No
HMF001701A	*BLM*	c.1642 C > T	p.Gln548*	P	recessive	LOF	no second hit	3	1.7	0	PTEN, RB1, TERT, TP53, chr7 gain, chr10 loss/LOH	Yes
HMF001787A	*MUTYH*	c.1178 G > A	p.Gly393Asp	P	recessive	LOF	no *MUTYH* signature, no second hit	3	1.5	0.1	EGFR, TERT, CDKN2A, chr7 gain, chr10 loss/LOH	No
HMF007027A	*SBDS*	c.258+2 T > C	p.? (splice)	P	recessive	LOF	no second hit	3	4.1	0.2	TERT, CDK4, EGFR, chr7 gain, chr10 los/LOH	Yes
HMF006506A	*FANCF*	c.484_485delCT	p.Leu162fs	P	recessive	LOF	no second hit	3	2.8	0.1	TERT, EGFR, PTEN, chr7 gain, chr10 loss/LOH	Yes
HMF000329A	*BLM* *MITF* *WRN*	c.1933C>T c.1255 G > A c.1105 C > T	p.Gln645* p.Glu419Lys p.Arg369*	P P P	Recessive dominant + recessive	LOF GOF LOF	no second hit	3	2.6	0.1	PTEN, TERT, CDKN2A, EGFR, chr7 gain, chr10 loss/LOH	No
HMF006895A	*HERC2* *MUTYH*	c.8002 G > C c.1138delC	p.Val2668Leu p.Ala382fs	VUS P	Dominant recessive	LOF LOF	no second hit no MUTYH signature, no second hit	3	4	0.1	ATRX, NF1, TERT, TP53, PTEN, chr7 gain, chr10loss/LOH	Yes
HMF003589A							MSI, (*MLH1* methylation?)	n/a	**216**	**11**	TERT, TP53, RB1	No
HMF001180A							MSI, somatic *MLH1*	n/a	**80**	**4**	TP53, CDKN2A, MLH1 (2x)	Yes

^1^Coding reference sequence, ^2^Protein reference sequence, ^&^14:102878068-17:29458864, *G* gain of function, *HGVS* Human Genome Variation Society, *LOF* loss of function, *WT* wildtype.

Classifications: *LP* likely pathogenic, *P* pathogenic, *VUS* variant of uncertain significance.

Inheritance: ^±^Risk factor for breast cancer, ^+^Risk factor for melanoma, ^−^Associated with glioblastoma.

^5^Out-of-frame skipping of the first 22 nucleotides of exon 8^86,87^ and observed with RNAseq, see supplemental Fig. 5.

^6^Splicing could not be proved with RNAseq data. This could be because the variant is lost in the tumor. The expert panel InSiGHT considers this variant as Likely pathogenic.

Tumor status: ^#^Somatic gain 17:29421945-29709134; complete gene is amplified in tumor, but amplification involved a large part of the chromosome (non-focal), HRD/HRP: homologous recombination deficient/proficient, MSI/MSS: microsatellite instability/stability.

Category: 1: causal event, 2: known predisposition gene but causality not demonstrated, 3: less likely to contribute to glioblastoma.

*NF1* complex event: NC_000014.8:g.pter_102878068delins[NC_000017.10:g.29458864_qter]/p.? (deletion of chr14pter_1st exon NF1).

*ATR* inv: NC_000003.11:g.142091960_142182304inv/p.? (inv exon 42-47 ATR + XRN1).

*BLM* deletion: NM_001287246.2(BLM):c.-4-1619_*16491del/p.

The 18 dominant inheritance PGVs were present in 10 different genes in 15 unique patients (16% of all patients). Six of these PGVs were in cancer predisposition genes (*ATR*, *CHEK2* (3x), *SDHA* and *MITF*) without an established association with familial glioblastoma. Interestingly, the majority, 12 PGVs in 10 patients, were in established cancer predisposition genes with a strong association with familial glioblastoma (*MSH6* (3x), *PMS2* (5x), *MSH2*, *NF1* and *BRCA1*) or with medulloblastoma (*SUFU*). Thus, the prevalence of known genetic predisposition to glioblastoma was 11% (10/92) in our unselected cohort, with additional candidates in another 5% of patients (5/92).

Among the 10 PGV carriers, the sex distribution was equal (5 females and 5 males), and no significant sex differences were observed. However, the median age at biopsy for the 10 PGV carriers (52 years; range: 19–72) was notably lower than that of the total cohort (61 years; range: 36–77). This difference was statistically significant, with a Mann–Whitney *U* test *p* value of 0.018.

### Genetic predisposition driving glioblastoma oncogenesis

As most predisposition genes involve tumor suppressors, all candidate causal events were assessed for second hit (somatic) events in the tumor data. PGVs with a second (somatic) event are considered causal for glioblastoma oncogenesis. For all six PGVs (*ATR*, *CHEK2* (3x), *SDHA* and *MITF*) without an established association with familial glioblastoma and for 10 out of 11 PGVs in recessive genes (*BLM* (4x), *ERCC3*, *MUTYH* (2x), *FANCF*, *SBDS* and *WRN*), no second (somatic) event (small variant or structural variant resulting in LOH) or matching mutational signature was detected in the tumor. Thus, those variants, except for possibly *BUB1B*, were unlikely to contribute to the development of glioblastoma in our cohort (category 3—see Table 1). Additionally, for three patients with PGVs in genes with a strong association with familial glioblastoma (*NF1*, *MSH6* and *MSH2*), no second (somatic) event or expected matching mutational signature was detected, indicating that for these variants the causality for tumorigenesis in these patients remains unclear (category 2—see Table 1).

**Fig. 1:**
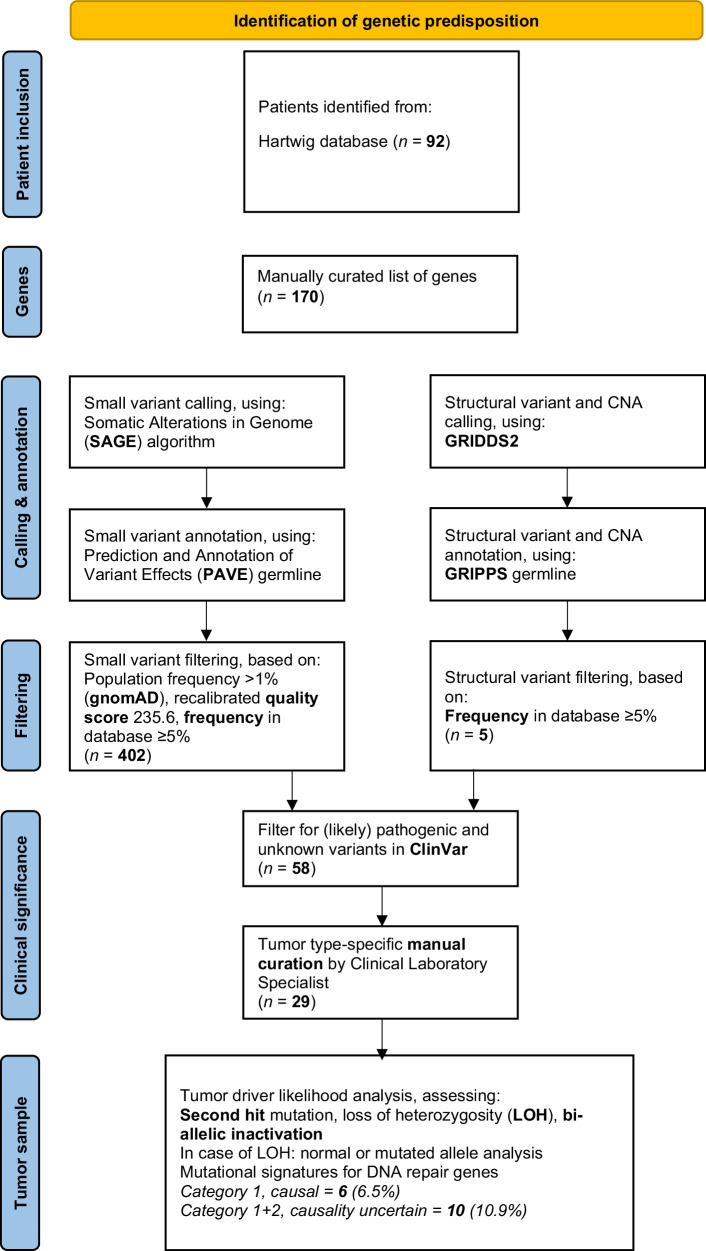
Flowchart methods, Word and Adobe Acrobat were used to create this figure.

Importantly, for the remaining nine PGVs that were identified in genes with a strong association with familial glioblastoma or medulloblastoma (*SUFU*, *MSH6* (2x), *PMS2* (5x) and *BRCA1*), a second (somatic) event and/or a matching mutational signature was identified in the tumor. These variants were present in seven different patients, resulting in a proven germline predisposition rate of 60% in the six patients with relevant PGVs (6/10). For the patient with a PGV in *SUFU*, accompanied by LOH of chromosome 10, the causal role of *SUFU* remains uncertain. The interpretability of this finding is hampered by the fact that chromosome 10 loss is a common event in glioblastoma. Nevertheless, somatic driver events in *SUFU* have been detected in the TCGA^35^ glioblastoma cohort (Supplementary Fig. 4; TCGA data), suggesting potential relevance in gliomagenesis, although the TCGA did not report recurrent *SUFU* homozygous deletions as a hallmark alteration. Of interest, two of these patients (age at biopsy 19 and 33) most likely have constitutional mismatch repair deficiency syndrome, since they each harbored two PGVs in *PMS2* and both were microsatellite instable with a high tumor mutational burden, without somatic driver mutations in any one of the four MMR genes (Table 1, Fig. 1).

### DNA damage response—significant role for mismatch repair (MMR) in glioblastoma

The known pathogenic predisposition variants in 10 patients could be divided into two main mechanisms. First, two patients had PGVs in genes involved in cell proliferation/survival (Ras/mitogen-activated protein kinase pathway; NF1 & Shh signaling pathway; *SUFU*). Second, eight patients had 10 PGVs in genes involved in the DNA damage response (*BRCA1*, *PMS2*, *MSH6* and *MSH2*). These included a patient showing LOH for *BRCA1* along with a homologous recombination deficiency (HRD) footprint (Fig. 1).

The majority of patients were thus found to harbor a PGV in one of the mismatch repair (MMR: MSH2, PMS2, MLH1, MSH6) genes (7 out of 11). By measuring microsatellite instability (MSI) based on WGS, we observed that, within the total cohort, seven patients had > 1.3 microsatellite Indels Per Mb (overall average 1.3, median 0.12) and six of these seven patients had ≥4 microsatellite Indels Per Mb (diagnostic cutoff of WGS handled by Hartwig Medical Foundation— see Fig. 1). For one of the seven patients with MSI no evidence for either germline or somatic mutations in any of the four MMR genes was found, this could be caused by MLH1 promoter hypermethylation (undetectable with WGS). For the remaining six patients with MSI, one patient with somatic loss of function of *MLH1* and five patients with germline loss of function of *MSH6* (2x) or *PMS2* (3x) matched with MSI. Interestingly, for all these patients, only a (somatic) driver event in the same MMR gene was detected (Table 1, Fig. 1).

**Fig. 2:**
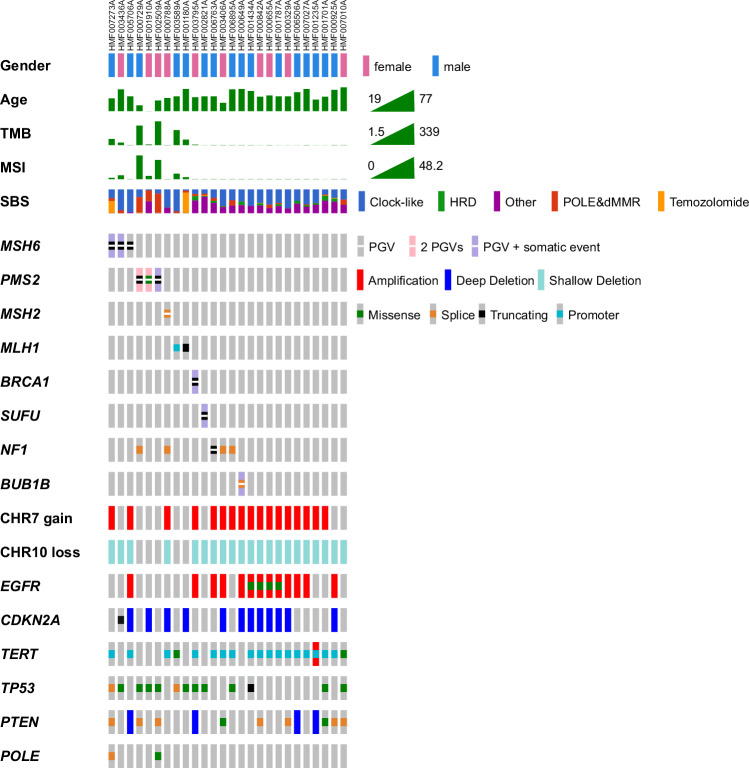
Significant role for mismatch repair (MMR) in glioblastoma. Gender; age at biopsy (years); tumor mutational burden (TMB); MSI (number of ms Indels per Mb) abundance of Cosmic single base substitution (SBS) signatures for dMMR&POLE/POLD1±dMMR (SBS6/9/10/14/15/20/21/26), Temozolomide (SBS11), HRD (SBS3) and Clock-like (SBS1); and germline/somatic aberrations are depicted for all patients present in Table 1. (patients with > 1.3 microsatellite Indels Per Mb and/or a PGV present). OncoPrinter (https://www.cbioportal.org/oncoprinter)^83–85^ and Adobe Acrobat were used to create this figure.

The percentage of patients with PGVs in MMR genes within this unselected glioblastoma cohort were compared to the percentage of patients with PGVs in MMR genes within other unselected cancer cohorts^20,36–38^ and the gnomAD v2.11 (non-cancer) cohort^39^. Although numbers remain small, a higher-than expected frequency of patients with glioblastoma carrying a PGV in MMR genes was seen, with the biggest difference for *MSH6* and *PMS2* (Fig. 2).

## Discussion

This study showed the germline predisposition in a cohort of 98 adult glioblastoma patients. In 11% of the patients, PGVs were observed in genes previously associated with familial glioblastoma; thus, these PGVs likely contributed to the oncogenesis of these unselected glioblastoma patients. PGVs were found in the following genes: *BRCA1*, *MSH6*, *PMS2, NF1* and *SUFU*. Furthermore, for nine PGVs in *SUFU*, *MSH6* (2x), *PMS2* (5x) and *BRCA1*, in six (seven including *SUFU*) different patients, causality was proven, since second (somatic) events and/or matching mutational signature were detected. Several of these PGVs were in predisposition genes that are increasingly important for (targeted) therapy selection, and for all findings, counseling by a clinical geneticist is indicated. Mismatch repair deficiency formed the main mechanism of the unselected cohort, with 7.1% of the patients harboring a PGV in one of the mismatch repair (MMR) genes, including five patients with microsatellite instability, where only a (somatic) driver event in the same MMR gene was detected.

The results of this study are unique in several aspects. First, no preselection based on personal and/or family history of malignant neoplasms was applied to the study cohort. Second, the pairing of both blood and tumor tissue samples allowed for verification of the causality of potentially interesting events. Third, since all patients underwent paired WGS testing combined with RNA sequencing (~60%), we were able to not only study point mutations (which is a limitation in most of the cancer predisposition research) but also copy number variations, structural variants, splice site variants (supplementary Fig. 5), mutational signatures, and expression.

We detected a number of PGVs in dominant and recessive genes without proven causality for glioblastoma, since the tumor sample analyses did not show second hit mutations in almost all of these cases. In the Netherlands, observed putative PGVs in dominant genes that do not match the tumor type (*ATR*, *CHEK2* (3x), *SDHA* and *MITF*) are normally not reported back to the patient, except if there is a matching personal and/or familial history. Unfortunately, in the current retrospective study design, we were not able to identify the pedigrees of the patients with PGVs, making further details of the inheritance pattern and possible consequences for family members impossible. In recessive genes, all 11 PGVs were monoallelic and considered low/no risk for cancer predisposition. Still, these variants potentially modified the genesis of the tumor as risk loci associated with susceptibility to glioblastoma. Unfortunately, our study lacked sufficient power to study these monoallelic PGVs in recessive genes in a statistically sound manner. Interestingly, in one patient with a PGV in *BUB1B*, the remaining wild-type allele was somatically lost due to a large deletion. The causality of the PGV in this recessive gene could not be demonstrated, although there is evidence for the role of *BUB1B* as a (pan-)cancer predisposing gene^40^, including glioblastoma^41^. When variants like these are identified, they are normally not reported back to the patient. Because these variants do not have any relevance for the patient nor for the patient’s family, except if there is consanguinity in the family, no genetic counseling and testing is recommended.

Currently, the international guideline of the European Association of Neuro-Oncology (EANO) on the diagnosis and treatment of diffuse gliomas of adulthood recommends genetic counseling in patients with ‘relevant germline variants or suspected hereditary cancer syndromes’^42^. This recommendation is based on low-level evidence (i.e., class IV, level C evidence) and did not specify which germline variants are considered relevant. The familial tumor syndromes associated with gliomagenesis named in this EANO guideline include neurofibromatosis type I, tuberous sclerosis, Turcot syndrome, Li-Fraumeni syndrome and Lynch syndrome. Other international guidelines of neuro-oncology or medical oncology societies lack recommendations on germline testing and genetic counseling of gliomas in adults^43,44^. However, the more recent EANO guideline on molecular testing of gliomas in adults recommends genetic counseling prior to germline testing, as for instance specific attention is paid to MMR gene deficiencies^45^. Yet, most of the PGVs found in our study are currently not tested for in most of the Dutch laboratories^46^.

As the use of comprehensive tumor genetic and genomic diagnostic tests continues to grow, the detection of PGVs is occurring more frequently than previously expected^36,41,47–52^. Notably, many of these PGVs are identified in individuals who do not meet current germline testing criteria and in cancers not traditionally associated with hereditary predisposition, particularly those historically classified as sporadic^36,52–54^. Furthermore, growing evidence suggests that family history alone is an unreliable predictor of germline risk^49,55^. Consequently, several have advocated for universal germline genetic testing, as current guideline-directed approaches may fail to identify up to 50% of patients harboring clinically actionable PGVs^47,51,53^. Consistent with these findings, our study also reveals a higher-than-expected prevalence of PGVs in patients with glioblastoma.

In addition, in our unselected cohort, many PGVs are identified in genes such as *BRCA1*, *MSH6*, *PMS2*, and *NF1*, which are crucial not only for germline follow-up but also for selecting appropriate therapies, particularly immune-based or targeted treatments, as observed in other tumor types. For example, melanoma, MMR-deficient colorectal cancer, and other non-colorectal MMR-deficient tumors have shown remarkable responses to immunotherapy^10–16,56^. While some glioblastoma patients exhibit long-term responses to immunotherapy, this treatment has shown limited efficacy in over 90% of unselected glioblastoma cases^57–60^. Among those who responded (partially or fully), most likely were patients with hypermutated tumors, possibly due to MMR deficiency or MMR deficiency + *POLE* defects^57,61–65^. Our findings indicate that most of these hypermutated tumors harbor a PGV in one of the MMR genes. Thus, comprehensive tumor genetic and genomic profiling for glioblastoma patients requires an integrated approach that facilitates appropriate referral to clinical geneticists.

This study has some limitations that have to be considered. First, this type of research cannot be done without making assumptions. Assumptions were not only made when defining the pathogenicity of variant^66–69^ but essentially every single step in our methods, e.g. variant calling, annotation, filtering, curation involved choices based on assumptions. Although these are based on generally accepted international standards^67–69^ changes over time based on progressive insights may impact outcomes. A second limitation is the relatively small sample size of cases and the lack of matching controls, which hampered statistically powered analyses of the PGVs. Third, due to consent and privacy regulation limitations, we were not able to assess the pedigrees of the patients with PGVs, making assessment of the inheritance pattern and possible consequences for family members impossible. Finally, the study lacked DNA methylation profiling, which would have enabled formal WHO CNS5 classification and identification of molecular subgroups^70^. Recent evidence indicates that adult MMR-deficient glioblastomas, including those with Lynch syndrome, may cluster with pediatric-type high-grade gliomas rather than conventional adult GBMs^71^, are often hypermutated, and may respond to immune checkpoint blockade. In our cohort, WGS revealed MSI, elevated TMB, and germline MMR mutations, supporting these observations and highlighting methylation profiling as a priority for future studies to guide immunotherapy selection.

To conclude, this study investigated the germline predisposition to glioblastoma in an average adult glioblastoma population. 11% of these patients had a PGV that (likely) predisposed them to the development of the glioblastoma, with potential associated therapy options. The results could guide clinicians who have to inform patients about broad molecular tests for personalized medicine and its associated putative germline findings, once current gene panels are adapted to these findings.

## Methods

### Study design and patient selection

For this retrospective germline analysis study, whole-genome sequencing data from the Hartwig Medical Foundation (Amsterdam, the Netherlands) database were used. All patients included in the database had previously provided informed consent for the reuse of their data, including germline data, for cancer research. Consent was obtained under a broad-use model, permitting access-controlled, publicly available use for academic research. Access to limited clinical information, as well as genome-wide germline and somatic data, was granted under Data Access Request DR-310. All samples were de-identified, and the linkage between patient and study identifiers was securely maintained solely within the originating hospitals. For this study, adult patients (aged 18 years or older) diagnosed with nervous system cancer (Disease Ontology ID: 3093), whose data were submitted to the database before November 1, 2023, were identified. From this cohort, only patients with gliomas classified as WHO grade 4 were included in the final analysis (*n* = 98).

Most of the patient data used in our retrospective germline analysis study were derived from two previously conducted clinical trials: CPCT-02 (NCT01855477; NL-OMON53030) and GLOW (NCT05186064; NL-OMON54315). Both studies were approved by their respective Medical Ethics Review Committees (METC), and all participants provided written informed consent prior to any procedures, in accordance with the Declaration of Helsinki. Family history and personal history of malignancies were not considered in either study.

In the GLOW study^9^, samples were collected from adult patients with histopathologically confirmed IDH wild-type glioblastoma at first recurrence, across twelve Dutch hospitals involved in neurosurgery and/or the treatment of glioblastoma.

In the CPCT-02 study^72^, samples were preferentially obtained from adult patients with metastatic disease prior to treatment initiation, across 41 academic, teaching, and general hospitals throughout the Netherlands.

### Whole-genome sequencing

All samples were sequenced at Hartwig Medical Foundation as per ISO-accredited diagnostic standards (ISO17025), as described previously^72^. Shortly, tumor samples with at least 20% tumor purity were deep-sequenced on Illumina Novaseq 6000 to an average depth of 90–100×. The blood control samples were sequenced to a depth of 30–35×. Somatic and germline variant calling was done using the open-source Hartwig WiGiTS toolset (https://github.com/hartwigmedical/hmftools v5_33). Also, tumor heterogeneity and presence of non-tumor cells in the tumor sample were computed (https://github.com/hartwigmedical/hmftools/tree/master/purple) and accounted for. For MSI detection in whole-genome sequencing (WGS) data, the pipeline contains a custom implementation of the MSIsensor tool^73,74^. The strategy for this germline and tumor analysis has been validated previously^8,36,75^ by the Pathology department (EN ISO 15189 accredited) at The Netherlands Cancer Institute. Furthermore, it has been since 2021 implemented as routine molecular diagnostics for solid cancer patients at The Netherlands Cancer Institute^76^ and by many other hospital in the Netherlands.

### Selection of relevant genes

Because of interpretation challenges and limited statistical power associated with the number of available patients compared to the vast search space of the genome, as well as the expected limited penetrance of individual genes, it was considered not feasible to perform a sufficiently powered genome-wide association study for analysis of variants that might be involved in glioblastoma predisposition. Hence, a manually curated list of known cancer-associated genes was created to first explore the potential involvement of candidate genes. As a basis, the reportable germline gene list used as part of the pan-cancer routine diagnostic analysis pipeline from Hartwig was used^36^. This gene panel is based on national guidelines^77^ and experience at the Netherlands Cancer Institute and was for this study expanded with genes from several other cancer predisposition gene panels: a germline driver catalog previously described and curated by Priestley et al.^72^, a subset of genes from the American College of Medical Genetics and Genomics (ACMG)^66^, the Hereditary Cancer Gene Curation Expert Panel from ClinGen^78^, the adult solid tumors cancer susceptibility panel created by National Health Service (NHS) and Genomics England^79^, and from the literature^20,30^. After comparing these different gene lists, a comprehensive list of 170 genes was generated for the current germline predisposition analysis. For all of these genes, the likely mechanism of action was determined as either oncogene or tumor suppressor gene (Supplementary Data 1).

### Small variant calling

Small variants include stop-gain mutations, frameshifts due to small insertions or deletions, in-frame deletions, in-frame insertions, missense mutations and splice site mutations. Within the standard pipeline workflow of Hartwig (https://github.com/hartwigmedical/pipeline5), small variants in both tumor and germline are called by the algorithm ‘Somatic Alterations in Genome’ (SAGE; v3.2) (https://github.com/hartwigmedical/hmftools/tree/master/sage). SAGE is a precise and highly sensitive caller for single nucleotide variants (SNVs), multiple nucleotide variants ≤32 base pairs (MNVs) and small insertions and deletions (InDels). In the standard data processing workflow of Hartwig, SAGE is given a panel containing the regions of genes of interest for germline analysis in a Browser Extensible Data (BED) format (Supplementary Data 1). For our selected gene panel, a custom BED file (https://github.com/MvOglow/germlineGBM.git) was created using the in-house tool HMF Gene Utilities (v1.1, https://github.com/hartwigmedical/hmftools/tree/master/gene-utils), which used the GENCODE coordinates for the Genome Reference Consortium Human Build 37 (GRCh37) definitions. All raw compressed reference-oriented alignment map (CRAM) files containing the aligned sequencing reads for the included patients were re-processed with SAGE using the default germline run parameters (v3.4; Supplementary Fig. 1) and these custom gene regions. Subsequently, this data was annotated and filtered using ‘Prediction and Annotation of Variant Effects’ (PAVE) germline (v1.6) (https://github.com/hartwigmedical/hmftools/tree/master/pave) using the default germline parameters (Supplementary Fig. 2).

Hereafter, variants annotated as having only synonymous canonical coding effects were removed from the output files. To reduce inclusion of common neutral population variants and potential false positives, additional filters were used next to the default SAGE filters: (1) variants with a Genome Aggregation Database (gnomAD; v2.1.1)^39^ non-cancer population cumulative frequency >1% were removed and classified as population variance; (2) germline variants with a low recalibrated quality score (see below) were removed and (3) germline variants with a frequency ≥5% in the Hartwig database (*n* = 5778, excluding the patients included in this study) were removed as these are likely population variants specific to the Dutch population. SAGE accounted for false positive calls or poor sensitivity by recalibrating the empirical base quality score provided by the sequencer. The ad-hoc cutoff based on these recalibrated Phred-scaled quality scores was determined using a density plot of the recalibrated Phred quality of all obtained variants for the included patients and set at 235.6 for variants to be included in further analyses (Supplementary Fig. 3).

### Structural variants and copy number variations calling

By default, structural variants (SVs) and copy number variations (CNVs) were called genome-wide by GRIDDS2 in the Hartwig pipeline^80^. After processing, this data was annotated and filtered by GRIPPS germline and stored in a SQL database (pipeline release v5.33). All SVs and CNVs within the regions defined in the BED file were obtained from the SQL database. Because gnomAD does not provide non-cancer population frequencies for SVs, the data were filtered based on the variant frequency within the Hartwig database (excluding the patients included in this study). All obtained SVs occurring in ≥5% of all other patients in the Hartwig database were excluded. Since the Hartwig databases contained 5778 patients, next to the patients included in this study, SVs occurring in ≥289 patients were discarded.

### Clinical significance

The clinical significance of variants was based on their annotation in ClinVar, a public archive of human genetic variants and interpretations of their significance to disease^81^. The main conclusions in our study were based on ‘pathogenic’ and ‘likely pathogenic’ variants. Variants of unknown significance were not studied. To direct the potential effect of these variants on the functional protein, the Ensembl Variant Effect Predictor was used^82^. All shortlisted variants were manually reviewed by a clinical laboratory geneticist (RK) to determine pathogenicity according to routine diagnostic procedures using the variant interpretation guidelines of the IARC, ACMG/AMP and ClinGen variant curation expert panels^67–69^. Likely-pathogenic (class 4) and pathogenic variants (class 5) were considered as PGVs. As a second step to assess the clinical significance of PGVs, tumor type-specific manual curation and tumor genome analysis were performed. The following subdivision was used: category 1 was causal events (gene associated with glioblastoma + matching tumor findings), category 2 was known predisposition genes but without demonstrated causality (gene associated with glioblastoma without matching tumor findings, or gene not associated but having matching tumor findings), and category 3 contained variants less likely to contribute to glioblastoma.

### Tumor sample analysis

**Fig. 3:**
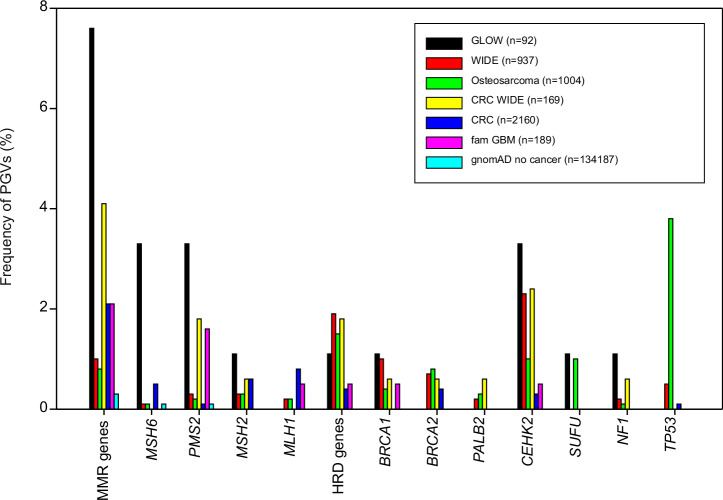
Frequency of pathogenic germline variants (PGVs) in the genes as described in the GLOW study versus other cohorts. CRC colorectal cancer^38^, CRC WIDE subgroup WIDE colorectal cancer patients^36^, fam GBM familial glioblastoma cohort^20^, GLOW current composite cohort, gnomad non-cancer reference cohort^39^, HRD genes homologous recombination deficiency genes (*BRCA1/2* & *PALB2*), dMMR genes deficient mismatch repair genes (*MSH6*, *PMS2*, *MSH2* & *MLH1*), MSI microsatellite instability, osteosarcoma^37^, WIDE metastatic cancer^36^ Excel and Adobe Acrobat were used to create this figure.

For tumor suppressor genes, the common model for pathogenicity is that both alleles of the gene become inactivated in the tumor. In case of germline predisposition, the second allele is typically inactivated by a second mutation or loss of heterozygosity (LOH, although epigenetic inactivation through methylation is also possible. Therefore, we assessed all candidate genes for somatic events, and, in case of LOH, determined if the normal or mutated germline allele was lost. In addition, we explored if any of the candidate genes were also a common somatic driver in glioblastoma patients, i.e., inactivated biallelically by somatic events. Finally, mutational signatures were studied for DNA repair genes. In case of splice site variants, RNA sequencing data (which was available for approximately 80% of the patients) was used to validate the impact of the variant at transcript levels. A graphic overview of the methods for the identification of predisposition can be found in Fig. 3.

### Statistics

Sociodemographic characteristics were compared by using chi-square test for categorical variables and t-test for continuous variables. In case of violation of the normality assumption, a non-parametric test was used for the continuous variables. Differences in median age between PGV carriers and non-carriers were assessed using the Mann–Whitney *U* test.

## Supplementary information

Supplementary Data 1

Supplementary Information

## Data Availability

The underlying research is partly facilitated by Hartwig Medical Foundation and the Center for Personalized Cancer Treatment (CPCT), which have generated, analyzed and made available data for this research. Data can be requested via [https://www.hartwigmedicalfoundation.nl/en/data/data-access-request/). Hartwig Medical Foundation is willing to share with external qualified researchers access to patient-level data and supporting clinical documents. These requests are reviewed and approved by an independent review committee on the basis of scientific merit. All data provided is anonymized to respect the privacy of patients who have participated in the study, in line with applicable laws and regulations.
